# Effect of dietary selenium from selenium-enriched kale sprout, selenomethionine, and sodium selenite on performance and selenium concentrations in the tissues of growing quails

**DOI:** 10.5713/ajas.20.0111

**Published:** 2020-05-12

**Authors:** Anut Chantiratikul, Orawan Arunsangseesod, Eakapol Wangkahart, Kwanyuen Leamsamrong, Piyanete Chantiratikul

**Affiliations:** 1 Division of Animal Science, Faculty of Technology, Mahasarakham University, Kantharawichai, Mahasarakham, 44150, Thailand; 2 Division of Fisheries, Faculty of Technology, Mahasarakham University, Kantharawichai, Mahasarakham, 44150, Thailand; 3 Department of Chemistry, Faculty of Science and Technology, Rajabhat Maha Sarakham University, Mueang, Mahasarakham 44000, Thailand; 4 Department of Chemistry and Center of Excellence for Innovation in Chemistry (PERCH-CIC), Faculty of Sciences, Mahasarakham University, Kantharawichai, Mahasarakham, 44150, Thailand

**Keywords:** Selenomethionine, Se-enriched Plant, Poultry, Glutathione Peroxidase

## Abstract

**Objective:**

This study aimed to determine the effect of dietary selenium (Se) from Se-enriched kale sprout (SeKS), selenomethionine (SeMet), and sodium selenite (SS) on performance, carcass characteristics and Se concentrations in the tissues, and to study the relationship between Se concentrations in muscle and feather in growing quails.

**Methods:**

The 320 quails (7 d of age) were divided into four treatments, according to a completely randomized design. The treatments were T1: control diet; T2, T3, and T4: control diets plus 0.2 mg Se/kg from SS, SeMet, and SeKS, respectively. The performance, carcass characteristics, and Se concentrations in the tissues of quails were determined.

**Results:**

The results indicated no effect (p>0.05) of Se supplementation on performance, carcass characteristics and glutathione peroxidase (GSH-Px) activity in breast muscle of quails. Supplemental Se from SS, SeMet, and SeKS increased greater (p<0.05) Se concentrations in breast muscle, liver, kidney, heart, and feather, compared to those of quails fed the control diet. Quails fed Se from SeMet had greater (p<0.05) Se concentrations in the tissues than quails fed Se from SeKS and SS. In addition, Se concentrations in breast muscle and feather of quails at 21 and 42-d-old were highly correlated (R^2^ 0.714 to 0.756) (p<0.05).

**Conclusion:**

Performance, carcass characteristics and GSH-Px activity in breast muscle of quails were not affected (p>0.05) by dietary Se supplementation. The Se from SeMet was more effective in increasing Se concentrations in the tissues of quails than Se from SeKS and SS. Feather Se concentrations of 21 and 42-d-old quails can be used for assessment of Se bioavailability of Se sources.

## INTRODUCTION

The crucial functions of selenium (Se) in poultry have been extensively documented. It is involved in immune responses, antioxidant defenses and other important physiological roles [1]. There are numerous reports that organic Se has greater bioavailable than inorganic Se in broilers, laying hens and quails, using Se concentrations in tissue and egg as an indicator [2,3]. Additionally, Sevcikova et al [4] found increasing Se concentrations in breast muscle and feather of broilers fed Se supplemental diets, compared to those of broilers fed the control diet. Couloigner et al [5] reported concurringly that breast muscle and feather Se concentrations were highly correlated in 21-d-old broilers. Thus, measurement of feather Se concentration could be used as a non-invasive procedure for investigation of the efficacy of Se sources for Se concentration in breast muscle. However, this suggestion should be scientifically confirmed.

Few trials have reported the responses of poultry fed supplemental organic Se from plants. Selenium from Se-enriched garlic (*Allium sativum* L.) and Se-enriched Chinese cabbage (*Brassica pekinensis* L.) was observed to improve Se status of broilers [6]. The effectiveness of Se from selenium enriched yeast (SeY) and Se-enriched mung bean sprout (*Vignaradiata*) was comparable in terms of egg Se concentration [7]. Dietary supplementation of Se-enriched Japanese radish sprouts (Raphanussativas) at 10 μg/kg increased serum Se level but resulted in variable Se concentrations in yolk and meat of laying hens [8]. On the other hand, Jiakui and Xiaolong [9] found the similar metabolic pathway of Se from Se-enriched malt (*Hordeumvulgare* L.) and sodium selenite (SS). Chantiratikul et al [10] demonstrated no different tissue Se concentrations of growing quails fed Se from SS and Se-enriched kale sprout (SeKS) cultivated in sand and watered by solution containing 60 mg Se from sodium selenate. Although, some variable outcomes had been found, those results indicated that Se from Se-enriched plants could possibly be used as an alternative Se source for poultry. Recently, hydroponically produced SeKS has been developed using SS as a Se source. Species of Se in SeKS were mostly present as organic forms [11]. The scientific reports found that the efficacy of Se from SeKS and SeY was comparable, but greater than that of Se from SS in laying quails [12], broilers [13], and laying hens [14]. Furthermore, Se from SeKS has been confirmed to be less toxic in laying hens [15] and in rats [16]. Nevertheless, there have not been any comparative studies on the effects of organic Se from SeKS and selenomethionine (SeMet) in growing quails. Therefore, the present trial aimed to compare the efficacy of dietary Se from SeKS, SeMet, and SS on productivity, carcass characteristics, meat quality, and tissue Se concentrations and to determine the relationship between Se concentrations in breast muscle and feather of growing quails.

## MATERIALS AND METHODS

### Animal care

The experimental procedures were approved by the Institutional Animal Care and Use Committee, Mahasarakham University (Approval No. IACUC-MSU-021/2019).

### The preparation of selenium-enriched kale sprouts

After being submerged in tap water for 15 h, the kale seeds (*Brassica oleracea* var. *alboglabra* L.) were planted into sponges and placed in plastic pots. The pots were completely covered for 3 d. Afterwards, the germinated seeds were illuminated with the light from a fluorescent lamp (36 W) and watered daily. The germinated seeds were then cultivated in Hoagland’s solution containing 30 mg Se from SS/L under the hydroponic system for 15 d. Finally, SeKS were gathered, washed, dried, ground, and stored [11].

### Dietary treatments

Three hundred and twenty, 1-d-old unsexed Japanese quails (*Coturnixcoturnix japonica*) were obtained from a commercial farm. The quails were fed the control diet for a week. On d 7, quails were randomly divided by their body weight (26.21 ±0.55 g) into four treatments. Each treatment consisted of four replicates and each replicate contained twenty quails in a Completely Randomized Design. The dietary treatments were T1: control diet; T2, T3, and T4: control diets plus 0.2 mg Se/kg from SS, SeMet (Excential Selenium4000, Orffa Additives BV, Werkendam, The Netherlands), and SeKS, respectively. Dietary inclusion level of Se at 0.2 mg/kg referred to EU regulations [17]. The control diet was prepared according to the nutrient requirement of growing quails [18], with no Se addition (Table 1). The diets were provided *ad libitum* to quails until slaughter at 42 d of age. Drinking water was freely available to quails at all time. The quails were reared in experimental cages (67×70×50 cm) placed under open housing system. Internal temperature was controlled at 28°C. The lighting was constantly maintained for 24 h in the cages.

### Data and sample collections

Feed consumption was recorded weekly. Body weight was determined at the beginning and at the end of each period, wk 2–3, and 4–6. Average daily gain (ADG) and feed conversion ratio (FCR) were estimated.

On d 21, 28, 35, and 42, two birds per replicate were ran domly selected and euthanized for breast muscle (pectoralis major and minor) and wing feather collections. The samples of breast muscle and feather were dried, ground and stored at −20°C.

On d 42, blood samples of eight birds in each treatment were drawn by puncturing the wing vein. The samples were placed into sterile test tubes and centrifuged at 3,000×g for 10 min. The serum was harvested and stored at −20°C. Afterwards, the birds were stunned and slaughtered for breast muscle (pectoralis major and minor) collection. The fresh samples of breast muscle were stored at −20°C.

At the end of the experiment, 8 birds from each treatment were randomly weighed, stunned, slaughtered, and exsanguinated. Wings, breast muscle, legs, heart, liver, gizzard, and abdominal fat were separated and weighed for determinations of carcass characteristics. The pH values of breast muscle were measured at 45 min and 24 h post-mortem by a portable pH meter (Model HI99163, Hanna Instruments, Padova, Italy). Color measurement of breast meat was performed at 45 min and 24 h post-mortem by a Chroma meter (Model CR-410, Konica Minolta Sensing Americans, Inc., New York, USA). The breast muscle, heart, liver, and kidney were sampled, dried, and ground.

### Analyses

The dietary treatment was determined for dry matter (DM, Method 934.01), crude protein (Method 976.05), ether extract (Method 920.39), crude fiber (Method 978.10), and ash (Method 942.05) according to AOAC [19]. Total Se concentrations in dietary treatments, dried tissues (breast muscle, heart, liver, and kidney), and feather were measured. Briefly, the weighed samples were wet digested by a mixture of 1.5 mL of 70% w/w nitric acid and deionized water in the digestion block at 100°C. When the solution had dried and cooled, 5 mL of HCl was added. The samples were then heated at 100°C for 10 min. After cooling the digest, deionized water was added into the volumetric flask to make up the reduced volume of the digest [20]. The Se concentrations in samples were analyzed with Atomic Absorption Spectrometer with a VGA-77 hydride generation unit (Agilent Technologies, Inc., Santa Clara, CA, USA).

Approximately 1 g of fresh breast muscle was homoge nized in 1 mL of 0.9% sodium chloride buffer using laboratory mortar with pestle. The supernatant was collected after centrifugation at 8,000 rpm for 10 min at 4°C and used for analysis. Glutathione peroxidase (GSH-Px) activity in breast muscle was measured according to the procedure of Paglia and Valentine [21].

### Statistical analysis

All experimental data was analyzed by analysis of variance technique appropriate for Completely Randomized Design [22]. The model used was: Y_ij_ = μ+T_i_+ɛ_ij_, where: Y_ij_ = observation, μ = population mean, T_i_ = diet effect (I = 1 to 4), and ɛ_ij_ = residual error. A cage was used as the experimental unit for productive performance measurements. Selected individual quails were considered the experimental unit for carcass characteristics, GSH-Px activity and tissue Se concentration, respectively. The differences among means of each parameter were compared by Duncan’s new multiple range test. Values of p<0.05 were considered significant. The relationships between Se concentrations in breast muscle and feather of 21 and 42-d-old quails were determined by linear regression analysis.

## RESULTS

The concentrations of Se in the control diet, control diets plus 0.2 mg Se from SS, SeMet, and SeKS were 0.25, 0.46, 0.43, and 0.42 mg/kg DM, respectively (Table 2).

### Effect of selenium sources on productivity and carcass characteristics of quails

The Se supplementations both in the form of inorganic (SS) and organic Se (SeMet and SeKS) did not change feed intake, ADG, FCR, carcass characteristics, and meat color of quails. However, the pH of meat at 24 h post-mortem increased (p<0.05) with Se supplementation, regardless of Se sources (Tables 3 to 5).

### Effect of selenium sources on glutathione peroxidase activity and Se concentrations in tissue of quails

Dietary Se supplementation did not influence GSH-Px activity in breast muscle of quails. Breast muscle, feather, and heart tissue Se concentrations of quails fed Se from SeMet were greater (p<0.05) than those of quails fed Se from SeKS and SS. Liver and kidney tissues Se concentrations of quails fed Se from SeMet and SeKS were similar (p>0.05), but greater (p<0.05) than those of quails fed the control diet and Se from SS (Table 6).

Quails that received Se from SeMet had greater (p <0.05) breast muscle and feather Se concentrations (Tables 7 to 8) at 21, 28, 35, and 42 d of age, compared to those of quails fed Se from SeKS and SS. Selenium from SeKS increased greater (p<0.05) Se concentrations in breast muscle than Se from SS (Table 7). However, Se concentrations in feathers of quails fed Se from SeKS and SS were not different (p>0.05) as presented in Table 8.

### Relationship of selenium concentrations in breast muscle and feather of quails

**Figure 1:**
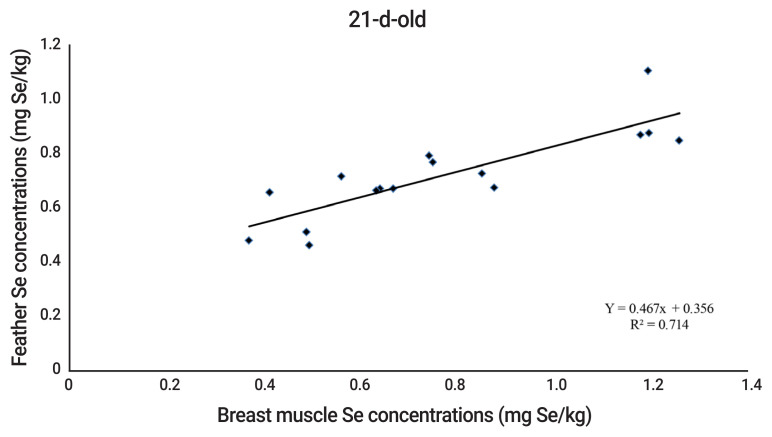
The relationship of selenium concentrations in breast muscle and feather of growing quails at 21-d-old.

**Figure 2:**
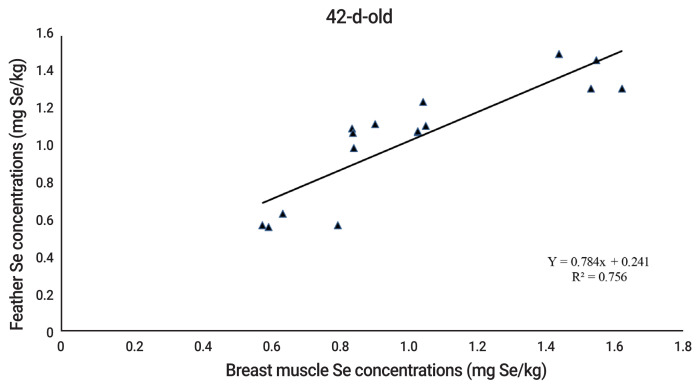
The relationship of selenium concentrations in breast muscle and feather of growing quails at 42-d-old.

The equations of relationship between Se concentrations in breast muscle and feather of quails were Y = 0.467x+0.356 and Y = 0.784x+0.241 (where Y is breast muscle Se concentration and x is feather Se concentration) for 21 and 42 d of age, respectively. The Se concentrations in breast muscle and feather were highly correlated with R^2^ = 0.714, and 0.756 for 21 and 42 d of age, respectively (Figures 1 to 2).

## DISCUSSION

The results demonstrated that Se supplementation in both inorganic and organic forms at 0.2 mg/kg did not change performance and carcass characteristics of growing quails. Chantiratikul et al [10] similarly found no impact of dietary Se supplementation form SeKS (0.2 to 1.0 mg/kg) on feed intake, performance, and carcass characteristics of quails. Trials in broilers also reported that Se sources from SS, SeY, SeKS, 2-hydroxy-4-methylselnobutanoic acid, and SeMet at 0.1 to 2.0 mg/kg diet did not alter feed intake and growth performance parameters [2,5], and carcass traits [13]. The Se concentration in the control diet was 0.25 mg/kg (Table 2), which met the Se requirement for quail (0.15 mg Se/kg) recommended by NRC [18]. Generally, performance of poultry reared in normal condition was not changed by supplementation of Se [6]. However, dietary Se supplementation improved productivity in heat-stressed poultry [23].

The results of this trial showed that meat color and meat pH at 45 min post-mortem were not altered by Se supplementation. However, pH of meat at 24 h post-mortem increased with Se supplementation (Table 5). Presently, there is no available published report on the effect of Se supplementation on meat quality in quail. Zhang et al [24] found that Se supplementation (0.3 mg/kg) from SS and SeMet did not improve meat color of 56-d-old offspring of broiler breeders. However, Wang et al [25] reported that broilers fed Se form SS and SeMet (0.15 mg/kg) resulted in increasing meat color value of breast muscle at 8 and 16 h post-mortem. Zhan et al [26] reported similarly that adding SS or SeMet in finishing pig diets caused an increasing trend of the pH value of loin muscle at45 min post-mortem in Se-treated groups. These results are similar to ours presented here. Generally, lower meat pH reduces the muscle protein ability to bind to water, causing shrinkage of myofibrils [26]. Therefore, the present results of the effect of Se supplementation on pH of meat at 24 h post-mortem reflected the observation that Se supplementation improved meat quality in quails. Nevertheless, additional research should be conducted for clarification of the effects of dietary Se addition on meat quality. Moreover, the above inconsistent findings might be due to different levels of Se supplementation, Se sources, Se species and animals used in the experiment.

GSH-Px is a selenoprotein enzyme, containing Se. It is an important antioxidant enzyme in animals. Our results found an insignificant effect of both inorganic (SS) and organic Se (SeMet and SeKS) supplementation on GSH-Px activity in breast muscle of quails (Table 6). Wang et al [25] have found similarly that GSH-Px activity in breast muscle of broilers was not affected by inorganic or organic Se supplementation. Those results could be explained by metabolic pathway of dietary Se. The metabolic route of Se is illustrated that both inorganic and organic Se can be effectively converted to hydrogen selenide (H_2_Se), which is used for selenoproteins synthesis such as GSH-Px [27].

The findings indicated that organic Se from SeMet and SeKS effectively increased Se concentrations in tissues of quails, compared to inorganic Se from SS (Table 6). Numerous reports have also demonstrated that organic Se has a better accumulative efficacy in tissues of poultry than inorganic Se [5,13,24]. The Se concentrations in breast muscle, heart tissue and feather of quails fed SeMet were significantly greater than those of quails fed SeKS. These results probably reflected a better efficiency of Se from SeMet compared to Se from SeKS. Additionally, breast muscle and feather Se concentrations (Tables 7 to 8) of quails at 21, 28, 35, and 42 d of age clearly confirmed the greater efficiency of Se from SeMet than those of SeKS. SeMet has been previously reported regarding its greater efficacy on Se depositions in tissues of broilers and in egg of laying hens than those of SeY [1]. The current trial evaluated the efficiency of Se from SeMet and SeKS using Se concentrations in tissues of growing quails as an index. The bioavailability of Se in animals mainly depends on SeMet content. SeMet can be built into body protein instead of methionine and allows Se accumulation in the tissues, resulting in greater Se concentration in muscle and other tissues [28]. The SeMet used in this trial contains a constant level of L-SeMet [28]. However, SeKS consists of SeMet and methyselenocysteine at 41% and 35%, respectively [11]. The greater SeMet content in SeMet than that in SeKS resulted in greater Se concentrations in tissues and feather of quails. The obtained results confirmed that Se from SeMet was more efficiently deposited in tissues of quails compared to Se from SeKS. The Se from SS had lower efficacy in tissue accumulation than Se from SeMet and SeKS. Additionally, kidney Se concentrations of quails fed both organic and inorganic Se seemed to be greater than other tissues Se concentrations (Table 6). Those results agree with the previous research in laying quails [12]. Ingested Se is generally used for selenoproteins synthesis after conversion to H_2_Se. However, unmetabolized Se will be mainly excreted via urine [27], resulting in greater Se accumulation in kidney than other tissues.

Naturally, pre-juvenile molting begins when the quails are 3-d-old. Juvenile body plumage is complete in about 30 days [29]. During this period, the second generation of feathers will be developed to replace the down feathers. Thus, Se content in the second generation of feathers depends on Se source and level in the diet. There is little information concerning relationship between Se in muscle or other tissues and feather of poultry. Quails fed drinking water with added SS or SeMet had the highest Se concentrations in the pancreas, followed by the down feathers, liver, and kidney [30]. Sevcikova et al [4] observed that Se concentrations in breast muscle and feather of 35-d-old broilers increased accordingly with dietary Se supplementation. Similarly, other researchers reported that the correlation coefficient between muscle and feather Se concentrations of 21 d-old broilers was 0.927 [5]. The present results concurringly found that Se concentrations in breast muscle and feather of 21 and 42-d-old quails were strongly related (R^2^ = 0.714 and 0.756 for 21 and 42-d-old, respectively). The results, therefore, reflect that feather Se concentration, instead of muscle Se concentration, can be used as an indicator for assessment of Se efficacy of Se sources in 21 and 42-d-old quails.

In conclusion, productive performance, carcass charac teristics, meat color, and GSH-Px activity in breast muscle of quails were not affected by dietary Se supplementation. Selenium from SeMet was more effective in increasing Se concentrations in the tissues of quails than Se from SeKS and SS. Feather Se concentrations of 21 and 42-d-old quails can be used for assessment of Se bioavailability of dietary Se sources.

## Figures and Tables

**Table 1:** Feed ingredient and chemical composition of the control diet1)

Items	% DM basis
Ingredients	
Corn	38.98
Rice bran	8.11
Soybean meal	37.18
Extruded soybean	9.70
Soybean oil	2.91
Dicalcium phosphate	1.07
Oyster shell meal	1.07
DL-methionine	0.27
L-lysine	0.27
Salt	0.22
Vitamin-mineral premix2)	0.22
Analyzed chemical composition	
Dry matter	93.48
Crude protein	23.38
Ether extract	7.36
Crude fiber	5.08
Ash	6.76
Available P by calculation	0.34
Calcium by calculation	0.86
ME (kcal/kg) by calculation	2,936

DM, dry matter; ME, metabolizable energy; Se, selenium; SS, sodium selenite; SeMet, selenomethionine; SeKS, Se-enriched kale sprout.

1) SS, SeMet, and SeKS (268.12 mg Se/kg) were thoroughly mixed in corn prior to adding to the diet to meet the treatment levels.

2) Vitamin–mineral premix provide (per kg diet): vitamin A, 10,000 IU; vitamin D_3_, 2000 IU; vitamin E, 12 mg; vitamin K_3_, 1.5 mg; thiamine, 1.5 mg; riboflavin, 4.0 mg; pantothenic acid, 10.0 mg; vitamin B_6_, 4.0 mg; vitamin B_12_, 0.02 mg; nicotinic acid, 3.0 mg; folic acid, 0.4 mg; biotin, 0.1 mg; Fe as iron and ion carbonate, 60.0 mg; Cu as copper sulfate, 8.0 mg; Mn as manganese oxide, 70.0 mg; Zn as zinc oxide, 50.0 mg; I as calcium iodide, 0.7 mg; and Co as cobalt sulfate; 0.5 mg.

**Table 2:** Selenium concentrations in the dietary treatments

Dietary treatments	Se (mg/kg DM)
Control diet	0.25
Control diet plus 0.2 mg Se/kg from sodium selenite	0.46
Control diet plus 0.2 mg Se/kg from selenomethionine	0.43
Control diet plus 0.2 mg Se/kg from Se-enriched kale sprout	0.42

Se, selenium; DM, dry matter.

**Table 3:** Effect of dietary selenium supplementation on productive performance of growing quails

Item	Dietary treatments1)				SEM	p-values

	T1	T2	T3	T4		
Feed intake (g DM/d)						
wk 2–3	10.55	10.60	10.43	10.45	0.32	0.998
wk 4–6	14.45	15.77	15.77	15.80	0.28	0.235
wk 2–6	12.89	13.70	13.64	13.66	0.34	0.818
Average daily gain (g/d)						
wk 2–3	4.74	4.85	4.83	4.87	0.05	0.864
wk 4–6	2.50	2.89	2.92	2.73	0.14	0.736
wk 2–6	3.40	3.67	3.68	3.59	0.14	0.891
Feed conversion ratio						
wk 2–3	2.21	2.17	2.15	2.13	0.05	0.953
wk 4–6	8.31	8.03	6.10	6.63	0.80	0.738
wk 2–6	5.87	5.68	4.52	4.83	0.55	0.796

Values are the mean of 4 replicates per treatment.

SEM, standard error of mean; DM, dry matter; Se, selenium; SS, sodium selenite; SeMet, selenomethionine; SeKS, Se-enriched kale sprout.

1) T1, control diet; T2, control diet plus 0.2 mg Se/kg from SS; T3, control diet plus 0.2 mg Se/kg from SeMet; T4, control diet plus 0.2 mg Se/kg from SeKS.

**Table 4:** Effect of dietary selenium supplementation on carcass characteristics of growing quails

Carcass characteristics	Dietary treatments1)				SEM	p-values

	T1	T2	T3	T4		
Live weight (g)	155.85	154.23	154.49	156.97	1.57	0.937
Carcass (%)	66.11	66.74	68.91	64.93	1.07	0.707
Carcass weight (% of live weight)						
Breast	18.91	18.83	19.68	18.65	0.28	0.599
Wings	6.17	6.01	5.58	5.73	0.16	0.575
Legs	15.52	14.67	15.66	14.83	0.31	0.603
Abdominal fat	0.77	0.87	0.87	0.91	0.09	0.702
Organ weight (% of live weight)						
Liver	1.93	1.97	1.80	1.83	0.09	0.905
Gizzard	1.52	1.48	1.59	1.45	0.03	0.399
Heart	0.81	0.78	0.92	0.91	0.04	0.601

Values are the mean of 8 replicates per treatment.

SEM, standard error of mean; Se, selenium; SS, sodium selenite; SeMet, selenomethionine; SeKS, Se-enriched kale sprout.

1) T1, control diet; T2, control diet plus 0.2 mg Se/kg from SS; T3, control diet plus 0.2 mg Se/kg from SeMet; T4, control diet plus 0.2 mg Se/kg from SeKS.

**Table 5:** Effect of dietary selenium supplementation on meat pH and meat color of growing quails

Item	Dietary treatments1)				SEM	p-values

	T1	T2	T3	T4		
pH						
pH 45 min	6.62	6.49	6.31	6.32	0.05	0.128
pH 24 h	5.58^b^	6.07^a^	6.07^a^	6.07^a^	0.06	0.001
Color2)						
45 min						
L*	44.62	43.70	43.36	44.98	0.39	0.460
a*	14.40	14.92	13.76	14.19	0.28	0.590
b*	3.93	3.96	4.58	4.39	0.30	0.864
24 h						
L*	47.62	45.93	45.96	45.94	0.75	0.846
a*	14.36	14.63	15.74	15.39	0.39	0.598
b*	5.30	5.26	5.68	4.89	0.24	0.756

Values are the mean of 8 replicates per treatment.

SEM, standard error of mean; Se, selenium; SS, sodium selenite; SeMet, selenomethionine; SeKS, Se-enriched kale sprout.

1) T1, control diet; T2, control diet plus 0.2 mg Se/kg from SS; T3, control diet plus 0.2 mg Se/kg from SeMet; T4, control diet plus 0.2 mg Se/kg from SeKS.

2) L*, relative lightness; a*, relative redness; b*, relative yellowness.

a,b Means in the same row with different letters are significantly different (p<0.05).

**Table 6:** The effect of dietary selenium supplementation on glutathione peroxidase activity in breast muscle and selenium concentrations in the tissues and feather of growing quails

Item	Treatments1)				SEM	p-values

	T1	T2	T3	T4		
GSH-Px (mU/mL)	28.69	29.55	31.56	31.02	1.95	0.971
Se concentration (mg/kg DM)						
Breast muscle	0.65^d^	0.86^c^	1.53^a^	1.03^b^	0.08	0.001
Feather	0.58^c^	1.07^b^	1.39^a^	1.12^b^	0.07	0.001
Liver	1.36^c^	2.16^b^	2.98^a^	2.67^a^	0.28	0.001
Kidney	3.43^c^	4.98^b^	6.08^a^	4.78^a^	0.16	0.001
Heart	0.92^c^	1.35^b^	1.98^a^	1.55^b^	0.10	0.001

Values are the mean of 8 replicates per treatment.

SEM, standard error of mean; GSH-Px, glutathione peroxidase; DM, dry matter; Se, selenium; SS, sodium selenite; SeMet, selenomethionine; SeKS, Se-enriched kale sprout.

1) T1, control diet; T2, control diet plus 0.2 mg Se/kg from SS; T3, control diet plus 0.2 mg Se/kg from SeMet; T4, control diet plus 0.2 mg Se/kg from SeKS.

a–d Means in the same row with different letters are significantly different (p<0.05).

**Table 7:** The concentrations of selenium in breast muscle (mg/kg dry matter) of growing quails fed different selenium sources at 21, 28, 35, and 42-d-old

Day-old	Treatments1)				SEM	p-values

	T1	T2	T3	T4		
21	0.44^d^	0.62^c^	1.20^a^	0.80^b^	0.07	0.001
28	0.49^d^	0.75^c^	1.33^a^	0.89^b^	0.08	0.001
35	0.56^d^	0.76^c^	1.34^a^	0.99^b^	0.07	0.001
42	0.65^d^	0.86^c^	1.53^a^	1.04^b^	0.08	0.001

Values are the mean of 8 replicates per treatment.

SEM, standard error of mean; Se, selenium; SS, sodium selenite; SeMet, selenomethionine; SeKS, Se-enriched kale sprout.

1) T1, control diet; T2, control diet plus 0.2 mg Se/kg from SS; T3, control diet plus 0.2 mg Se/kg from SeMet; T4, control diet plus 0.2 mg Se/kg from SeKS.

a–d Means in the same row with different letters are significantly different (p<0.05).

**Table 8:** The concentrations of selenium in the feather (mg/kg dry matter) of growing quails fed different selenium sources at 21, 28, 35, and 42-d-old

Day-old	Treatments1)				SEM	p-values

	T1	T2	T3	T4		
21	0.52^c^	0.68^b^	0.92^a^	0.73^b^	0.04	0.001
28	0.56^c^	0.84^b^	1.05^a^	0.82^b^	0.05	0.001
35	0.61^c^	1.04^b^	1.46^a^	0.95^b^	0.08	0.001
42	0.58^c^	1.07^b^	1.39^a^	1.12^b^	0.07	0.001

Values are the mean of 8 replicates per treatment.

SEM, standard error of mean; Se, selenium; SS, sodium selenite; SeMet, selenomethionine;SeKS, Se-enriched kale sprout.

1) T1, control diet; T2, control diet plus 0.2 mg Se/kg from SS; T3, control diet plus 0.2 mg Se/kg from SeMet; T4, control diet plus 0.2 mg Se/kg from SeKS.

a–c Means in the same row with different letters are significantly different (p<0.05).

## References

[1] Surai PF, Kochish II, Fisinin VI, Velichko OA (2018). Selenium in poultry nutrition: from sodium selenite to organic selenium sources. J Poult Sci.

[2] Yoon I, Werner TM, Butler JM (2007). Effect of source and concentration of selenium on growth performance and selenium retention in broiler chickens. Poult Sci.

[3] Chantiratikul A, Chinrasri O, Chantiratikul P (2008). Effect of sodium selenite and zinc-L-selenomethionine on performance and selenium concentrations in eggs of laying hens. Asian-Australas J Anim Sci.

[4] Sevcikova S, Skrivan M, Dlouha G, Koucky M (2006). The effect of selenium source on the performance and meat quality of broiler chickens. Czech J Anim Sci.

[5] Couloigner F, Jlali M, Briens M, Rouffineau F, Geraert P, Mercier Y (2015). Selenium deposition kinetics of different selenium sources in muscle and feathers of broilers. Poult Sci.

[6] Seo TC, Spallholz JE, Yun HK, Kim SW (2008). Selenium-enriched garlic and cabbage as a dietary selenium source for broilers. J Med Food.

[7] Chinrasri O, Chantiratikul P, Thosaikham W (2009). Effect of selenium-enriched bean sprout and other selenium sources on productivity and selenium concentration in eggs of laying hens. Asian-Australas J Anim Sci.

[8] Hossain MS, Afrose S, Takeda I, Tsujii H (2010). Effect of selenium-enriched Japanese radish sprouts and *Rhodobacter capsulatus* on the cholesterol and immune response of laying hens. Asain-Australas J Anim Sci.

[9] Jiakui L, Xiaolong W (2004). Effect of dietary organic versus inorganic selenium in laying hens on the productivity, selenium distribution in egg and selenium content in blood, liver and kidney. J Trace Elem Med Biol.

[10] Chantiratikul A, Chinrasri O, Pakmaruek P, Chantiratikul P, Thosaikham W, Aengwanich W (2011). Responses of growing Japanese quails that received selenium from selenium enriched kale sprout (*Brassica oleracea* var. *alboglabra* L.). Biol Trace Elem Res.

[11] Maneetong S, Chookhampaeng S, Chantiratikul A (2013). Hydroponic cultivation of selenium-enriched kale (*Brassica oleraceavar*. *alboglabra* L.) seedling and speciation of selenium with HPLC-ICP-MS. Microchem J.

[12] Chinrasri O, Chantiratikul P, Maneetong S, Chookhampaeng S, Chantiratikul A (2013). Productivity and selenium concentrations in egg and tissue of laying quails fed selenium from hydroponically produced selenium-enriched kale sprout (*Brassica oleracea* var. *alboglabra* L.). Biol Trace Elem Res.

[13] Chantiratikul A, Pakmaruek P, Chinrasri O (2015). Efficacy of selenium from hydroponically produced selenium-enriched kale sprout (Brassica oleracea var. alboglabra L.) in broilers. Biol Trace Elem Res.

[14] Chantiratikul A, Chinrasri O, Chantiratikul P (2018). Effect of selenium from selenium-enriched kale sprout versus other selenium sources on productivity and selenium concentrations in egg and tissue of laying hens. Biol Trace Elem Res.

[15] Chantiratikul A, Borisuth L, Chinrasri O (2016). Evaluation of the toxicity of selenium from hydroponically produced selenium-enriched kale sprout in laying hens. J Trace Elem Med Biol.

[16] Leamsamrong K, Tongjaroenbuangam W, Maneetong S, Chantiratikul A, Chinrasri O, Chantiratikul P (2019). Physicochemical contents, antioxidant activities, and acute toxicity assessment of selenium-enriched Chinese kale (*Brassica oleracea* var. *alboglabra* L.) seedlings. J Chem.

[17] EFSA (2013). Scientific opinion on the safety and efficacy of L-selenomethionine as feed additive for all animal species. EFSA J.

[18] National Research Council (1994). Nutrient requirements of poultry.

[19] Helrichv K (1990). AOAC International Official methods of analysis of the AOAC International.

[20] Kapolna E, Fodor P (2006). Speciation analysis of selenium enriched green onions (*Allium fistulosum*) by HPLC-ICP-MS. Microchem J.

[21] Paglia DE, Valentine WN (1967). Studies on the quantitative and qualitative characterization of erythrocyte glutathione peroxidase. J Lab Clin Med.

[22] SAS Instiute (1988). SAS/STAT user’s guide: release.

[23] Habibian M, Sadeghi G, Ghazi S, Moeini MM (2015). Selenium as a feed supplement for heat-stressed poultry: a review. Biol Trace Elem Res.

[24] Zhang L, Wang YX, Zhou Y, Zheng L, Zhan XA, Pu QH (2014). Different sources of maternal selenium affect selenium retention, antioxidant status, and meat quality of 56-day-old offspring of broiler breeders. Poult Sci.

[25] Wang YX, Zhan XA, Zhang XW, Wu RJ, Yuan D (2011). Comparison of different forms of dietary selenium supplementation on growth performance, meat quality, selenium deposition, and antioxidant property in broilers. Biol Trace Elem Res.

[26] Zhan XA, Wang M, Zhao RQ, Li WF, Xu ZR (2007). Effects of different selenium source on selenium distribution, loin quality and antioxidant status in finishing pigs. Anim Feed Sci Technol.

[27] Rayman MP, Infante HG, Sargent M (2008). Food-chain selenium and human health: spotlight on speciation. Br J Nutr.

[28] Rovers M, Segers L, Du Laing G (2016). Effect of dietary selenium source on selenium deposition in broiler muscle tissue.

[29] Summers DDB (1972). Pterylography, plumage development and moult of Japanese quail Coturnix c. japonica in captivity. Int J Avian Sci.

[30] Anan Y, Ohbo A, Tani Y, Hatakeyama Y, Yawata A, Ogra Y (2012). Distributionand metabolism of selenite and selenomethionine in the Japanese quail. Metallomics.

